# Development of an online resource for recruitment research in clinical trials to organise and map current literature

**DOI:** 10.1177/1740774518796156

**Published:** 2018-08-31

**Authors:** Anna Kearney, Nicola L Harman, Anna Rosala-Hallas, Claire Beecher, Jane M Blazeby, Peter Bower, Mike Clarke, William Cragg, Sinead Duane, Heidi Gardner, Patricia Healy, Lisa Maguire, Nicola Mills, Leila Rooshenas, Ceri Rowlands, Shaun Treweek, Akke Vellinga, Paula R Williamson, Carrol Gamble

**Affiliations:** 1 North West Hub for Trials Methodology Research, University of Liverpool, Liverpool, UK; 2 Clinical Trials Research Centre, University of Liverpool, Liverpool, UK; 3 Health Research Board – Trials Methodology Research Network, National University of Ireland, Galway, Ireland; 4 ConDuCT-II Hub for Trials Methodology Research, University of Bristol, Bristol, UK; 5 North West Hub for Trials Methodology Research, Population Health Sciences, University of Manchester, Manchester, UK; 6 Northern Ireland Methodology Hub, Queen’s University Belfast, Belfast, UK; 7 MRC Clinical Trials Unit at UCL, London, UK; 8 Health Research Board – Trials Methodology Research Network, College of Medicine, Nursing and Health Sciences, National University of Ireland, Galway, Ireland; 9 Health Services Research Unit, University of Aberdeen, Aberdeen, UK; 10 School of Medicine, National University of Ireland, Galway, Ireland

**Keywords:** Recruitment, randomised controlled trial, clinical trial, accrual, barriers and facilitators, recruitment interventions

## Abstract

**Background:**

Recruiting the target number of participants within the pre-specified time frame agreed with funders remains a common challenge in the completion of a successful clinical trial and addressing this is an important methodological priority. While there is growing research around recruitment, navigating this literature to support an evidence-based approach remains difficult. The Online resource for Recruitment Research in Clinical triAls project aims to create an online searchable database of recruitment research to improve access to existing evidence and to identify gaps for future research.

**Methods:**

MEDLINE (Ovid), Scopus, Cochrane Database of Systematic Reviews and Cochrane Methodology Register, Science Citation Index Expanded and Social Sciences Citation Index within the ISI Web of Science and Education Resources Information Center were searched in January 2015. Search strategy results were screened by title and abstract, and full text obtained for potentially eligible articles. Studies reporting or evaluating strategies, interventions or methods used to recruit patients were included along with case reports and studies exploring reasons for patient participation or non-participation. Eligible articles were categorised as systematic reviews, nested randomised controlled trials and other designs evaluating the effects of recruitment strategies (Level 1); studies that report the use of recruitment strategies without an evaluation of impact (Level 2); or articles reporting factors affecting recruitment without presenting a particular recruitment strategy (Level 3). Articles were also assigned to 1, or more, of 42 predefined recruitment domains grouped under 6 categories.

**Results:**

More than 60,000 records were retrieved by the search, resulting in 56,030 unique titles and abstracts for screening, with a further 23 found through hand searches. A total of 4570 full text articles were checked; 2804 were eligible. Six percent of the included articles evaluated the effectiveness of a recruitment strategy (Level 1), with most of these assessing aspects of participant information, either its method of delivery (33%) or its content and format (28%).

**Discussion:**

Recruitment to clinical trials remains a common challenge and an important area for future research. The online resource for Recruitment Research in Clinical triAls project provides a searchable, online database of research relevant to recruitment. The project has identified the need for researchers to evaluate their recruitment strategies to improve the evidence base and broaden the narrow focus of existing research to help meet the complex challenges faced by those recruiting to clinical trials.

## Background

The challenges associated with completing a successful clinical trial are numerous and varied. However, a common problem lies in the recruitment of participants. Successfully recruiting the pre-specified number of participants within the planned time frame is difficult and can negatively impact all stakeholders.^1,2^ Since the reports by McDonald et al.^1^ and Bower et al.^2^ in the mid-2000s, there has been significant investment in infrastructure^3^ to support clinical trials in the United Kingdom. However, the challenge of achieving adequate recruitment remains.^4,5^

The importance of overcoming recruitment difficulties was identified as the top priority for methodological research, in a Delphi survey of Clinical Research Collaborative registered Clinical Trials Units in the United Kingdom in 2011–2012.^6^ A lower than expected recruitment rate can delay the identification and availability of effective treatments by decreasing the power of the study, increasing time and costs required for trial delivery and in some cases leading to early termination of studies. In 2011, 19% of trials on the National Library of Medicine registry were terminated early citing accrual problems and an estimated 48,027 people were enrolled in trials that were unlikely to meaningfully answer the primary research question due to insufficient number of participants.^7^

Lower than expected recruitment may be due to several factors, and strategies are often put in place during trials to help improve the recruitment rate. As a result, the approaches used are responsive and their impact might not be assessed.^8–10^

As recruitment to time and target is a challenge for many trials, efficient management of the recruitment literature would allow trialists and methodology researchers to access and use relevant information to improve recruitment to studies, assess the methods that have been used to evaluate recruitment strategies and identify uncertainties that warrant further research. Currently, navigating the published literature for evidence on recruitment strategies is difficult and time consuming. CONSORT guidelines do not require published reports of randomised controlled trials to describe recruitment methods. Recruitment information may be poorly reported including only the minimum amount of information to comply with the guidelines. Consequently, most trial reports do not provide a useful resource for identifying recruitment interventions. Recruitment issues might be more likely to be reported if the trial is stopped early, thereby identifying barriers rather than facilitators to recruitment. Furthermore, even if a trial report contains information on the effects of a specific recruitment strategy, identifying such information in the tens of thousands of reports of trials published each year would be an overwhelming task.

The ORRCA project (Online resource for Recruitment Research in Clinical triAls) aims to create an online resource of research to help trialists and others to identify interventions relevant to specific recruitment challenges. We describe the development of the ORRCA online database and summarise the included literature in this article.

## Methods

The development of the ORRCA database involved three key steps: identification of relevant literature, mapping of this literature to pre-specified recruitment research domains and extraction of relevant data from included studies. These steps are described below.

### Search strategies and identification of literature

A librarian assisted with the development of database-specific search strategies (Online Supplementary Material, Supplementary File 1) based on those used by Treweek et al.^11,12^ The search strategies were agreed by the Study Management Group, made up of the co-applicants on this research project. The following databases were searched during January 2015, with no restriction on language or publication date:

- Cochrane Database of Systematic Reviews and Cochrane Methodology Register as components of the *Cochrane Library*, www.cochranelibrary.com
- MEDLINE via Ovid
- Scopus (including EMBASE)
- Education Resources Information Center, CSA
- Science Citation Index Expanded, ISI Web of Science
- Social Sciences Citation Index, ISI Web of Science

Additional references were found through hand-searching systematic reviews of nested randomised evaluations of recruitment interventions (Online Supplementary Material, Supplementary File 1).

### Inclusion and exclusion criteria

Studies were included if they reported or evaluated recruitment strategies, interventions or methods and if the full text of their report was available in English.

As well as studies of recruitment to randomised trials, articles reporting recruitment to other health research designs such as cohort studies, observational studies, surveys, focus groups and biobank donations were included as a source of transferable knowledge and ideas. However, the search strategy was not focused on these areas.

A full list of exclusion criteria is available within Supplementary File 1 in the Online Supplementary Material.

### Identification and training of volunteer reviewers

Screening of the identified materials was done by a team of volunteer reviewers identified through the University of Liverpool Clinical Trials Research Centre, the Hub for Trials Methodology Network Recruitment Working Group and the Health Research Board Trials Methodology Research Network. Reviewers had methodological research experience, were provided with written guidance and expected to attend a training session, in-person or by teleconference.

### Development of a schema of recruitment research domains

**Figure 1.**
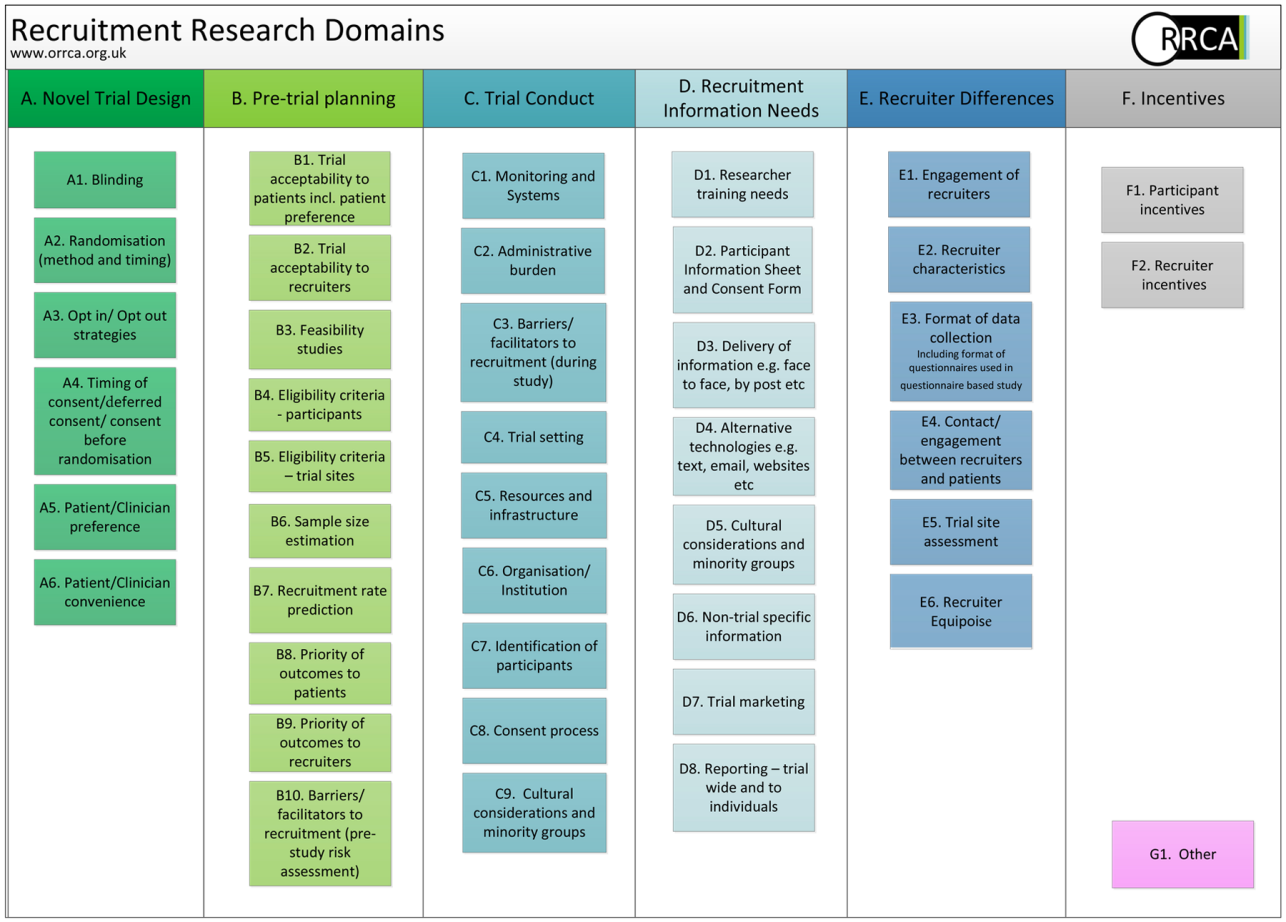
Conceptual framework for recruitment research domains.

A taxonomy of recruitment research themes was developed to categorise literature and map research efforts. The taxonomy drew on existing work by Caldwell et al.,^8^ who broadly grouped 37 trials of recruitment strategies that they had identified for a systematic review into four categories: novel trial design; incentives; provision of trial information; and recruiter differences. An additional two categories, ‘trial conduct’ and ‘pre-trial activities’, (Figure 1) were added along with a breakdown of domains within each category. The taxonomy was presented to the Hub for Trials Methodology Network Recruitment Working Group and the Study Management Group for agreement before being piloted, and was reviewed throughout the project to ensure relevance to the emerging literature.

### Screening and data extraction

Articles were screened by title and abstract across the team of reviewers. Ten percent of abstracts were independently checked for eligibility and rescreened by a different reviewer if more than 10% of errors were identified. The full text of all potentially eligible articles was then obtained and assigned a primary reviewer. A secondary reviewer was assigned to 50% of the articles to ensure consistency across inclusion criteria, research domains and level of evidence. Inter-rater reliability scores were not calculated due to the number of abstracts and full text articles. Queries or disagreements were resolved through discussion with a third reviewer. Eligible articles were categorised into each relevant recruitment domain and according to one of the following categories of evidence:

*Level 1*. Systematic reviews, nested randomised controlled trials and case-control studies evaluating the effects of recruitment strategies. This includes recruitment to hypothetical trials and quasi-randomised studies.
*Level 2*. Studies that report recruitment strategies without an evaluation of impact. This includes informal evaluations such as level of recruitment before and after a strategy is applied.
*Level 3*. Articles that report possible factors affecting recruitment but do not present a particular recruitment strategy. This includes studies evaluating reasons for participation or non-participation, and lessons learnt from trials.

Included articles were not assessed for the quality of the evidence or risk of bias, a task left to the database users due to the scale of the review.

Details of eligible articles and their categorisation were uploaded onto a free, publicly accessible website (www.orrca.org.uk) throughout the literature review process. Additional pre-specified information for each eligible article was extracted. This information was used to populate search filters that would allow users of the ORRCA website to refine searches and identify research relevant to different populations and health conditions (Supplementary Table S1 in the Online Supplementary Material). A free text search box on the website homepage allows users to search across all article titles, abstracts and extracted data.

Articles initially coded as ‘other’ (G1) were reviewed for the possible creation of new recruitment domains, re-coding into existing domains or inclusion in the G1 domain.

### Analysis

Analysis of articles was conducted in SAS 9.3 and SAS 9.4. Website use statistics for September 2016–May 2017 were obtained using Google analytics. Search criteria and number of searches were obtained from the ORRCA database, which anonymously records all searches performed in order to evaluate uptake of the resource.

## Results

**Figure 2.**
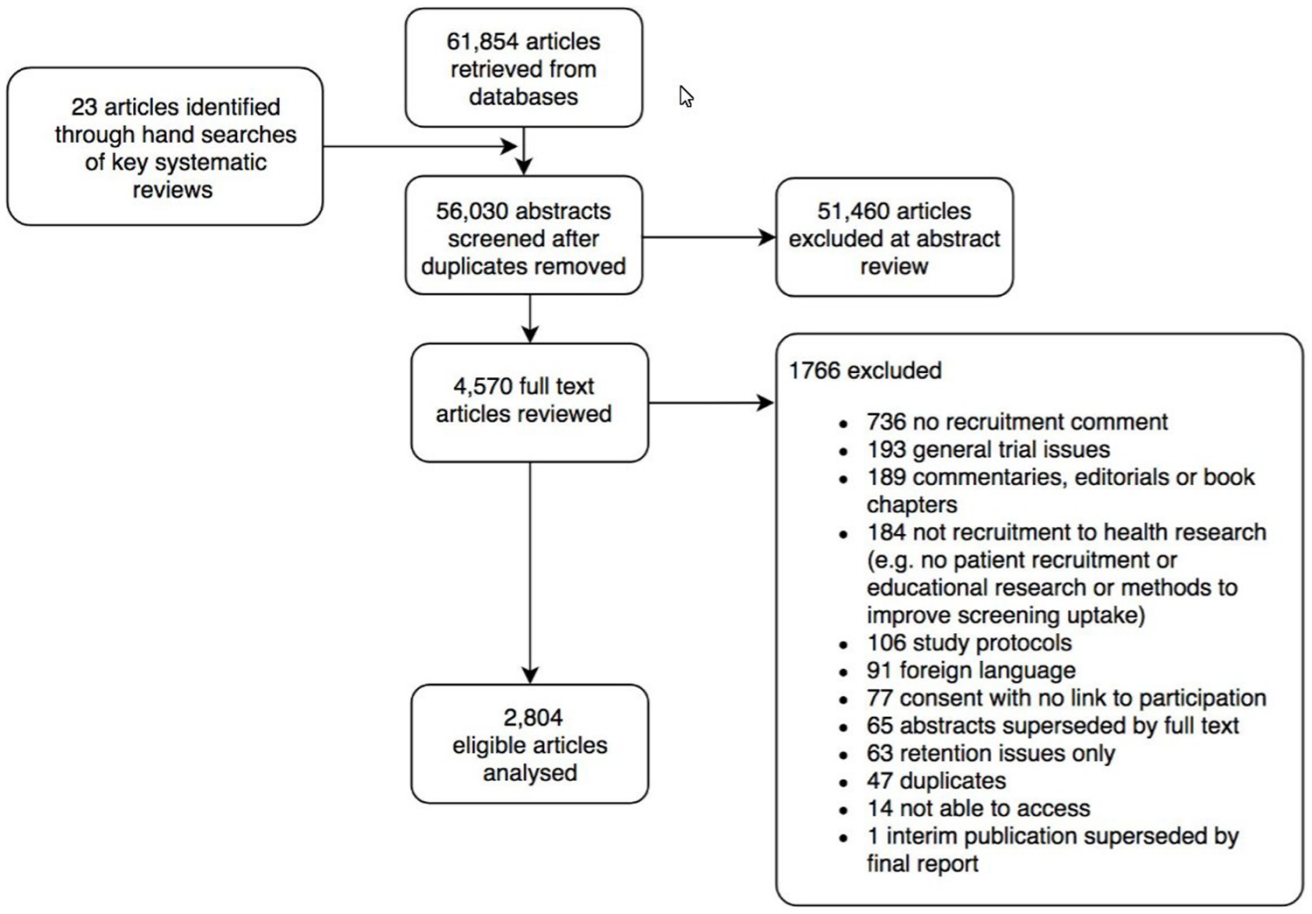
ORRCA literature search.

More than 60,000 articles were identified through electronic databases with a further 23 articles identified through hand searches. Following removal of duplicates, 56,030 titles and abstracts were screened and 4570 full text articles were reviewed. A total of 2804 articles were included in the online database (Figure 2).

**Figure 3.**
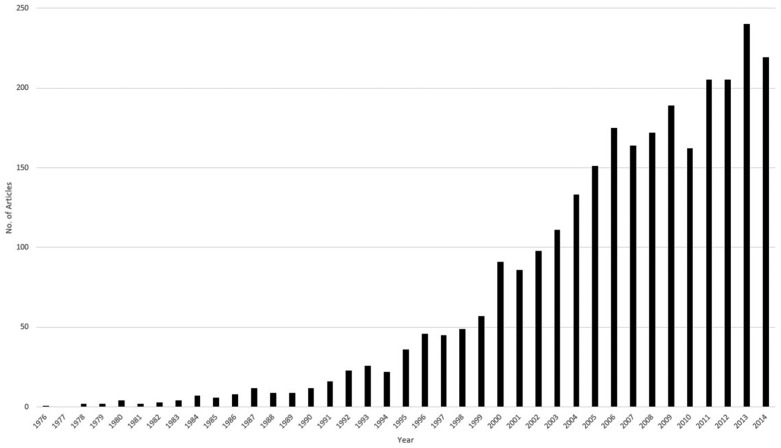
Year of publication (n = 2804).

Included articles covered all Health Research Categorisation System^13^ topic areas (Online Supplementary Material, Supplementary Table S2), with cancer studies (25%) and mental health studies (13%) being the most frequent. Articles covered recruitment research across the world although the majority reported recruitment within North America (53%) or Europe (25%) with only 2% reporting information from Africa and 1% from South America. Over half of the articles described recruitment of participants aged between 18 and 60 years (51%) and one-third focused on participants older than 60 years (35%). There were relatively few studies addressing recruitment of children under 16 years (12%) or aged between 16 and 18 years (7%). The number of articles per year generally increased over time (Figure 3) and the majority were published in journals focussed on clinical trials, cancer, epidemiology and family practice (Online Supplementary Material, Supplementary Table S3).

A total of 1883 articles were categorised as evidence ‘level 3’ (67%), with only 160 (6%) categorised as ‘level 1’ and 761 (27%) as ‘level 2’.

Studies could be relevant to more than one recruitment domain and on average each paper contributed 2.5 domains, with 7060 domains recorded across the 2804 included articles (Table 1). The most commonly populated domains were Barriers and Facilitators identified in Trial Conduct (37%) and Pre-trial Planning (17%), Identification of Participants (26%) and Cultural and Minority Considerations (16%) (Online Supplementary Material, Supplementary Table S4).

**Table 1.** Frequency of domains within domain categories and across evidence levels.

Domain category			Evidence level					
	Overall (2804 articles)		Level 1 (160 articles)		Level 2 (761 articles)		Level 3 (1883 articles)	
	Count of domains	% (n = 7060)	Count of domains	% (n = 336)	Count of domains	% (n = 2161)	Count of domains	% (n = 4563)
A: Novel trial design	216	3.1	38	11.3	78	3.6	100	2.1
B: Pre-trial planning	1517	21.5	23	6.9	272	12.6	1222	26.8
C: Trial conduct	3336	47.3	65	19.4	1073	49.7	2198	48.2
D: Recruitment information needs	1111	15.7	154	45.8	479	22.2	478	10.5
E: Recruiter differences	607	8.6	28	8.3	152	7.0	427	9.4
F: Incentives	273	3.9	28	8.3	107	5.0	138	3.0
Total Median [IQR] domains per article	7060 2 [1,3]	100	336 2 [1,2]	100	2161 3 [2,4]	100	4563 2 [1,3]	100

**Figure 4.**
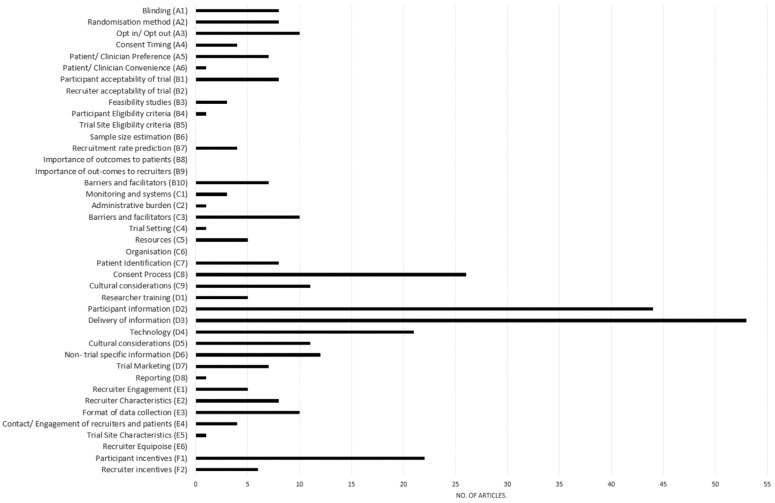
Distribution of Recruitment Domains in Level 1: All articles categorised as evaluating the effectiveness of strategies or interventions (n = 160).

Articles included in evidence level 1 were most frequently categorised in domain category D (Recruitment and Information Needs) with 53 evaluating the method of information delivery (33%) and 44 (28%) evaluating the content and format of participant information (Figure 4). No articles evaluated the effects of interventions or strategies related to sample size estimation, the importance of outcomes, organisation/institutional factors or recruiter equipoise. Articles in evidence levels 2 and 3 were most often categorised in the ‘trial conduct’ domain category describing barriers and facilitators to recruitment.

### Website use

The online database was launched on 1 September 2016 and is accessible via the website www.orrca.org.uk. In the first 9 months since the launch, 1058 searches of the database have been undertaken with 1139 users visiting the website from 18 countries (Online Supplementary Material, Supplementary Figure S1 and Table S5).

The most popular method of searching the database and filtering the literature was through the recruitment domains (35%) followed by use of the free text search box on the homepage (23%) (Online Supplementary Material, Supplementary Table S6). The most popular search filters addressing trial design or context were health area (5%), recruitment approach (3%), health intervention type (3%), age (3%), recruitment setting (3%) and host design (3%). The most frequently searched domains were B7 (Recruitment Rate Prediction) and C3 (Barriers and Facilitators) (Online Supplementary Material, Supplementary Table S7). However, it is important to note that during this analysis period, ORRCA was used to support a systematic review of recruitment rate prediction models and a priority setting exercise for evaluating recruitment interventions (The PRioRiTy study).^14,15^

## Discussion

Recruitment research in clinical trials remains a priority. The large number of articles identified for inclusion in the ORRCA database and the extensive effort needed to identify them, together with the subsequent use of the website, reinforce the need for a resource to enable trialists to access the findings of relevant recruitment research. Mapping the research included in the database highlights a continued emphasis on evaluating information for participants in clinical trials and a paucity of evidence in other areas, in particular, the impact of outcome choice, trial site factors and recruiter equipoise on recruitment.

Most domains identified in the eligible studies were contained within the Trial Conduct category, reflecting the large number of case reports (evidence levels 2 and 3) of recruitment methods and interventions. Several of the frequent domains were broad, such as Barriers and Facilitators (B10 and C3) and Trial Acceptability to Patients (B1). The relatively large number of articles on methods for engaging cultural and ethnic minorities (C9) can be explained by the large representation of North American research and the National Institute of Health’s legislation mandating the inclusion of women and minorities in research studies.^16,17^

Despite the increasing quantity of recruitment research, the evidence base for effective recruitment strategies remains weak. A number of topics have not been considered but we recognise that some of these will be difficult to assess through nested randomised studies or embedded studies and will require evaluation through other research methods. Domains such as Organisation/Institution (C6) and Sample Size Estimation (B6) feature more prominently in articles categorised as evidence levels 2 and 3, suggesting that trialists are aware of their importance and are discussing their impact on recruitment but without doing high-level evaluations to investigate them. In contrast, Recruiter Equipoise (E6), Trial Site Eligibility (B5), Trial Site Assessment (E5) and the Importance of Outcomes to both recruiters (B9) and patients (B8) were rarely identified in the eligible literature. While there has been significant emphasis on giving greater consideration to the choice of outcomes in clinical trials, including the development and selection of appropriate core outcome sets,^18,19^ it appears that the impact of the choice of outcomes on recruitment is not yet a subject of published research, although future studies may be planned.^20^

An online survey of directors of Clinical Trial Units^21^ highlights a wide range of approaches used to improve recruitment and the lack of evaluation of most of these. Systematic reviews of nested randomised evaluations of recruitment interventions^8,11,22^ have shown the challenges of identifying relevant literature, the inability of individual studies to demonstrate evidence for benefit^11^ and the variability in interventions. These issues make it difficult for studies to perform meta-analyses.^8,11^ It is perhaps not surprising, therefore, that, despite their relatively frequent evaluation within nested randomised trials and systematic reviews, optimising the consent process and trial participant literature continues to feature in the top 10 priorities for recruitment research.^14,15^

More research is needed to strengthen the evidence base.^9,23,24^ However, concerns over the perceived complexity of embedding methodological research studies, uncertainty as to how potential funders will view the work, the impact on the host trial and concerns about the capacity of the trial team to support them^24^ may all be limiting their uptake despite the guidance and support offered from initiatives such as the Studies Within A Trial^25,26^ and Medical Research Council’s Systematic Techniques for Assisting Recruitment to Trials initiative.^27–29^ The new initiative from the National Institute for Health Research Health Technology Assessment programme to provide up to £10,000 for embedded studies linked to bids^30^ will help within the United Kingdom. Practical guidance on how to embed methodological research into host studies has also recently been published.^31^

Recruitment methods and information can affect subsequent patient retention, an area where there is also a paucity of evidence for effective practices.^32^ Given concerns over the additional work needed to embed methodological studies in host trials, exploration of the relationship between recruitment and retention interventions is warranted to identify opportunities to run studies that evaluate both recruitment and retention interventions at the same time.

The ORRCA database will be updated annually to ensure it remains a useful resource for addressing recruitment challenges in trials, can support new systematic reviews and identify areas for future methodological research. Authors and funding bodies are also encouraged to submit recently published or ongoing studies through the website to avoid unnecessary duplication of effort.

### Strength and limitations

Comprehensive searches of multiple databases and the engagement of multiple reviewers have allowed a large-scale literature review. Although inclusion required access to an English language publication, only 2% of potentially eligible full text articles were excluded due to the prohibitive costs of translation and it is uncertain how many of these would have eventually met the inclusion criteria. Furthermore, our extensive search strategies, together with the characteristics of the eligible articles, demonstrate that the online database and mapping exercise are internationally relevant.

The scale of the ORRCA project contributed to limitations within the coding approach. Reviewers needed methodology research experience, received training and written guidance and were advised to take an inclusive approach to coding domains. However, domain coding was complex given the number of papers reviewed, the poor reporting and the lack of formalisation of recruitment strategies within case reports. Users of the database are therefore encouraged to act as additional reviewers and to recommend changes or coding of additional domains through the ‘contact us’ section of the website.

Individual articles were assigned all relevant recruitment domains without any weighting in order to create a simple and effective search functionality. Consequently, it is not possible to ascertain the primary recruitment topic addressed in each article. Articles categorised within evidence level 1 (with the exception of systematic reviews) were allocated fewer domains on average, so this problem largely impacts on articles at evidence levels 2 and 3 and, in particular, on case reports.

Although our search strategies focused on recruitment to clinical trials, a wider approach was taken during the review process. Articles describing recruitment to other health research designs such as cohort studies, biobanks and questionnaires were included to incorporate insights that might be transferable to randomised trials. However, the database does not contain a comprehensive review of recruitment strategies for non-randomised studies, and is limited to articles identified through the search strategy that we adopted.

### Future research

Mapping of the eligible recruitment research identifies unexplored areas which warrant further evaluation. However, even frequently evaluated topics, such as patient consent information, still need further research due to the current lack of conclusive evidence, which points to the need to improve both the focus and rigour of future evaluations.

## Conclusion

The ORRCA project involved undertaking an extensive review of the recruitment literature. Mapping and analysis of the 2804 articles in the initial version of the online database (www.orrca.org.uk) provides insight into existing research efforts and highlights topics for future collaborative research, promoting the reduction of waste in both methodology research and clinical trials. By successfully engaging methodology researchers from across the United Kingdom and Ireland, we have demonstrated that large-scale collaborative methodological projects are possible.

## Supplemental Material

796156_supp_mat – Supplemental material for Development of an online resource for recruitment research in clinical trials to organise and map current literature Click here for additional data file. Supplemental material, 796156_supp_mat for Development of an online resource for recruitment research in clinical trials to organise and map current literature by Anna Kearney, Nicola L Harman, Anna Rosala-Hallas, Claire Beecher, Jane M Blazeby, Peter Bower, Mike Clarke, William Cragg, Sinead Duane, Heidi Gardner, Patricia Healy, Lisa Maguire, Nicola Mills, Leila Rooshenas, Ceri Rowlands, Shaun Treweek, Akke Vellinga, Paula R Williamson and Carrol Gamble in Clinical Trials

## Supplemental Material

ct-17-0208-File006 – Supplemental material for Development of an online resource for recruitment research in clinical trials to organise and map current literature Click here for additional data file. Supplemental material, ct-17-0208-File006 for Development of an online resource for recruitment research in clinical trials to organise and map current literature by Anna Kearney, Nicola L Harman, Anna Rosala-Hallas, Claire Beecher, Jane M Blazeby, Peter Bower, Mike Clarke, William Cragg, Sinead Duane, Heidi Gardner, Patricia Healy, Lisa Maguire, Nicola Mills, Leila Rooshenas, Ceri Rowlands, Shaun Treweek, Akke Vellinga, Paula R Williamson and Carrol Gamble in Clinical Trials

## Supplemental Material

ct-17-0208-File007 – Supplemental material for Development of an online resource for recruitment research in clinical trials to organise and map current literature Click here for additional data file. Supplemental material, ct-17-0208-File007 for Development of an online resource for recruitment research in clinical trials to organise and map current literature by Anna Kearney, Nicola L Harman, Anna Rosala-Hallas, Claire Beecher, Jane M Blazeby, Peter Bower, Mike Clarke, William Cragg, Sinead Duane, Heidi Gardner, Patricia Healy, Lisa Maguire, Nicola Mills, Leila Rooshenas, Ceri Rowlands, Shaun Treweek, Akke Vellinga, Paula R Williamson and Carrol Gamble in Clinical Trials
