# Supplementary material for: Development of an online resource for recruitment research in clinical trials to organise and map current literature
Source: Clin Trials. 2018 Aug 31;15(6):533–42. doi: 10.1177/1740774518796156 (PMC6236587; doi:10.1177/1740774518796156)
Supplement: 796156_supp_mat – Supplemental material for Development of an online resource for recruitment research in clinical trials to organise and map current literature [file 796156_supp_mat.pdf]

Supplementary File 1.

- **Search Strategies**
- **Exclusion Criteria**
- **Systematic Reviews hand searched for additional papers**

Search Strategies and Exclusion Criteria

CMR (Cochrane Library Online) – archived in July 2012
#1 "accrual and sample size":kw or "attitudes to trials":kw or "informed consent":kw
#2 (participat* or recruit* or enrol* or select*) near/8 (trial* or research or study):ti or (participat* or recruit* or enrol* or select*) near/8 (trial* or research or study):ab
#3 (#1 OR #2)

Cochrane Database of Systematic Reviews (Cochrane Library Online)
#1 "accrual and sample size":kw or "attitudes to trials":kw or "informed consent":kw
#2 (participat* or recruit* or enrol* or select*) near/8 (trial* or research or study):ti or (participat* or recruit* or enrol* or select*) near/8 (trial* or research or study):ab
#3 (#1 OR #2)

MEDLINE via Ovid
1. Patient Selection/
2. ((participat* or recruit$*or enrol*) adj4 trial?).tw
3((Participant* or subject* or patient* or volunteer*) adj4 trial*)
4.((Participant* or subject* or patient* or volunteer*) adj4 selection)
5.((Participant* or subject* or patient* or volunteer*)adj4 recruit*)
6. 1 or 2 or 3 or 4 or 5
7 Informed Consent/
8. informed consent.tw
9. consent adj5 recruit*
10. 7 or 8 Or 9
11. exp Clinical Trial as Topic/
12. Research Subjects/
13. (trial? or study or studies or research).tw.
14. 11 or 12 or 13
15. 6 or (10 and 14)
16. Research Support, NIH, Extramural.pt.
17. Research Support, NIH, Intramural.pt.
18. Research Support, Non US Gov't.pt.
19. Research Support, US Gov't, Non PHS.pt.
20. Research Support, US Gov't, PHS.pt.
21. recruit* adj4 random*
22. recruitment.ab. /freq=2
23. participation.ab. /freq=2
24. research.tw.
25. or/16-24
26. randomized controlled trial.pt.
27. controlled clinical trial.pt.
28. random$.ab.
29. 26 or 27 or 28
30. humans.sh.

31. 29 and 30
32. comment.pt.
33. editorial.pt.
34. 31 not (32 or 33)
35. 15 and 25 and 34

SCOPUS (including EMBASE)

1. TITLE (participat*) OR TITLE(recruit*) OR TITLE(enrol*) OR TITLE(enter*) OR TITLE (entry) OR TITLE (accru*)
2. TITLE(trial?) OR TITLE(study)
3. 1 and 2
4. TITLE-ABS (select W/3 participants) or TITLE-ABS (select W/3 patients) or TITLE-ABS (select W/3 controls) or TITLE-ABS (select W/3 subjects) or TITLE-ABS (select W/3 volunteers)
5. ABS (recruit*)
6. ABS (participat*)
7. TITLE-ABS(research)
8. 5 or 6 or 7
9. 4 and 8
10. TITLE-ABS (informed consent) or TITLE-ABS (consent) or TITLE-ABS (consent process*) or TITLE-ABS (consent procedure?)
11. TITLE-ABS (patient W/2 information)
12. TITLE-ABS (patient W/2 leaflet)
13. TITLE-ABS (patient W/2 booklet)
14. TITLE-ABS (patient W/2 video)
15. TITLE-ABS (patient W/2 website)
16. 11 OR 12 OR 13 OR 14 OR 15
17. TITLE-ABS (participant W/2 information)
18. TITLE-ABS (participant W/2 leaflet)
19. TITLE-ABS (participant W/2 booklet)
20. TITLE-ABS (participant W/2 video)
21. TITLE-ABS (participant W/2 website)
22. 17 OR 18 OR 19 OR 20 OR 21
23. TITLE-ABS (subject W/2 information)
24. TITLE-ABS (subject W/2 leaflet)
25. TITLE-ABS (subject W/2 booklet)
26. TITLE-ABS (subject W/2 video)
27. TITLE-ABS (subject W/2 website)
28. 23 OR 24 OR 25 OR 26 OR 27
29. 16 OR 22 OR 28
30. INDEXTERMS (feasibility AND study) or INDEXTERMS (pilot AND project)
31. INDEXTERMS (Clinical AND Trial)
32. TITLE-ABS (trial?) OR TITLE-ABS (study) OR TITLE-ABS (studies) OR TITLE-ABS (research)
33. 10 and (29 OR 30 OR 31 OR 32)
34. 3 OR 9 OR 33

35. INDEXTERMS (randomised AND controlled AND trial)
36. TITLE-ABS (random*)
37. INDEXTERMS (major AND clinical AND study)
38. 35 OR 36 OR 37
39. INDEXTERMS (nonhuman)
40. DOCTYPE (ed)
41. 39 OR 40
42. 38 AND NOT 41
43. 34 AND 42
44. DOCTYPE (le)
45. 43 AND NOT 44

Science Citation Index Expanded and Social Sciences Citation Index, ISI
TS=(recruitment same "clinical trial") or TS=(recruitment same "clinical trials") or TS=(recruitment same "controlled trial") or TS=(recruitment same "controlled trials")

ERIC
(recruit* or participat*) and ((clinical trial*) or (controlled trial) or randomi*)

Exclusion Criteria

- Studies that evaluate understanding or recall of consent without linking this to participation
- Studies that do not recruit patients e.g. audit of services, recruitment of clinicians for continuing education
- Studies exploring interventions to improve education within schools. (Health research conducted within schools or other educational settings is included.)
- Studies recruiting patients to screening or vaccination programmes or evaluating methods to improve the uptake of screening/ vaccination. Articles reporting recruitment to vaccine trials or trials of screening methods (e.g. blood tests vs endoscopy) or trials comparing screening to no screening are included.
- Studies exploring informed consent and decision making for surgery or standard medical procedures.
- Studies only reporting issues with retention such as the return of questionnaires within a wider study.
- Commentaries, editorials, book chapters and protocols.
- Studies assessing selection bias without a link to recruitment strategies or recruitment factors.

Systematic reviews hand searched for additional papers:

1. Auster J, Janda M. Recruiting older adults to health research studies: A systematic review. Australasian Journal on Ageing. 2009;28(3):149-51.
2. Barnard K, Dent L, Cook A. Systematic literature review of predicting recruitment to clinical trials [abstract]. Clinical Trials [Internet]. 2009; 6(5):[550 p.]. Available from: http://onlinelibrary.wiley.com/o/cochrane/clcmr/articles/CMR-14822/frame.html.
3. Caldwell P, Craig J, Hamilton S. Strategies for recruitment to RCTs: a systematic review of controlled trials and observational studies. International Clinical Trials Symposium: improving health care in the new millenium; 2002 Oct 21-23; Sydney, Australia [Internet]. 2002:[34-5 pp.]. Available from: http://onlinelibrary.wiley.com/o/cochrane/clcmr/articles/CMR-4685/frame.html.
4. Foy R, Parry J, Duggan A, Delaney B, Wilson S, Lewin-van den Broek N, et al. How evidence based are recruitment strategies to randomized controlled trials in primary care? Experience from seven studies. Family Practice. 2003;20(1):83-92.
5. Huynh L, Johns B, Liu SH, Vedula SS, Li TJ, Puhan MA. Cost-effectiveness of health research study participant recruitment strategies: A systematic review. Clinical Trials. 2014;11(5):576-83.
6. Lai GY, Gary TL, Tilburt J, Bolen S, Baffi C, Wilson RF, et al. Effectiveness of strategies to recruit underrepresented populations into cancer clinical trials. Clinical Trials. 2006;3(2):133-41.
7. Treweek S, Lockhart P, Pitkethly M, Cook JA, Kjeldstrom M, Johansen M, et al. Methods to improve recruitment to randomised controlled trials: Cochrane systematic review and meta-analysis. Bmj Open. 2013;3(2).
8. UyBico SJ, Pavel S, Gross CP. Recruiting vulnerable populations into research: A systematic review of recruitment interventions. Journal of General Internal Medicine. 2007;22(6):852-63.
9. Watson JM, Torgerson DJ. Increasing recruitment to randomised trials: a review of randomised controlled trials. BMC Medical Research Methodology. 2006;6:34.
10. Mapstone J, Elbourne D, Roberts I. Strategies to improve recruitment to research studies. Cochrane Database of Systematic Reviews. 2007(2).
11. Raftery J, Bryant J, Powell J, Kerr C, Hawker S. Payment to healthcare professionals for patient recruitment to trials: systematic review and qualitative study. Health Technol Assess [Internet]. 2008; 12(10):[1-128 pp.]. Available from: http://onlinelibrary.wiley.com/o/cochrane/clcmr/articles/CMR-14367/frame.html.

Notes: *Uybico et al* was initially included in level 1 but was amended to level 2 following review and discussion with the third reviewer. The additional articles identified through hand searches of this review have remained in the database.
