# Supplementary material for: Development of an online resource for recruitment research in clinical trials to organise and map current literature
Source: Clin Trials. 2018 Aug 31;15(6):533–42. doi: 10.1177/1740774518796156 (PMC6236587; doi:10.1177/1740774518796156)
Supplement: ct-17-0208-File007 – Supplemental material for Development of an online resource for recruitment research in clinical trials to organise and map current literature [file ct-17-0208-File007.pdf]

Supplementary File 2: Additional tables and figures

- **Table S1: Additional data abstracted for database search filters.**
- **Table S2: Detailed Characteristics of the Cohort**
- **Table S3: Top Ten Journals publishing recruitment research**
- **Figure S1: Total website users, sessions and searches from September 2016 and May 2017**
- **Table S4: Distribution of individual domains against evidence categories**
- **Table S5: Countries accessing ORRCA website between September 2016 and May 2017**
- **Table S6: Analysis of search terms used within the ORRCA database between September 2016 and May 2017**
- **Table S7: Frequency of searches for individual recruitment domains**

Table S1: Additional data abstracted for database search filters.

Article details	Further information
Abstract	Article identifiers.
Journal	
Volume	
Issue	
Pages	
Year	
DOI	
**Overview of Research**	
Recruitment domain(s)	Key recruitment theme(s) contained within the article.
Evidence type	Articles have been assigned one of three categories based on the type of recruitment evidence.<br>**Level 1:** The formal assessment of the effectiveness of a recruitment strategy through a nested randomised control trial, a case control or a systematic review.<br>**Level 2**: Information on the use of a recruitment strategy without formal evaluation. This includes informal evaluations such as level of recruitment before and after a strategy is applied.<br>**Level 3:** Concepts, observations or information that may be used to inform future recruitment strategies. This includes lessons learnt from trials and reasons for participation/ non participation.
Recruitment intervention	A brief summary of the recruitment intervention/ strategy that is being evaluated. Please note this category has only been completed for articles formally evaluating recruitment strategies.
Summary of findings	A brief summary of the findings of the recruitment research. Please note this category has only been completed for articles formally evaluating recruitment strategies.
Research location	All continents where the research was conducted.
Funder	Type of funding received for the research
Research methods	Research methods used to evaluate recruitment such as a nested randomised control trial, survey of participants or a case report sharing lessons learnt from a health research study.
Research outcomes	The outcomes used to evaluate recruitment. For these database 'Recruitment rate' is defined as numbers recruited per month or year. 'Representativeness' looks at the diversity of research participants compared to the general population. 'Numbers recruited' also includes percentage of screened patients that were recruited.

Timing within host study	Often recruitment research is conducted for or during a health research study. This category indicates when the recruitment research was undertaken in relation to the health research e.g. during the feasibility, during an ongoing study.
**Context**	
Host design	The design of the health research study to which patients are being recruited e.g. cohort study, interview or a survey. Randomised controlled trials have been split into different categories. RCT is used for parallel trials or where there is no further information on the trial design. Where more specific information is available trials have been categorised as Cluster RCT, Crossover RCT, Factorial RCT or Patient Preference.
Recruitment approach	Type of approach used to recruit participants. A 'direct' approach is defined as approaching patients who are known or highly likely to meet eligibility criteria. Indirect approaches such as newspaper advertising target broader audiences of whom only some will be eligible.
Recruitment setting	The healthcare setting where patients are recruited. For mailing lists we have taken into account where the lists were generated and/ or where final consent conversations were undertaken.
Health area	The Health Research Classification System (HRCS) category for the patient's condition and/ or host research.
Health description	This free text field gives more specific information on the health area e.g. HIV.
Health intervention type	The type of intervention patients receive during the health research e.g. a drug, surgical procedure or medical device
Health intervention aim	The aim of the health intervention e.g. treatment or prevention
Health intervention setting	The healthcare setting where the treatment intervention is given and / or monitored.
Age	The age range of patients. Where possible we have based this on the patient eligibility criteria. However, in some cases we have had to use the baseline characteristics of participants to identify likely categories.
Gender	Gender of patients recruited. Where articles report more than one host study all relevant categories have been chosen. Where articles do not specify we will assume the study recruits both genders.

Blinding	Information on who was blind to the treatment allocation. The gender of patients recruited to the study. Where articles report more than one study all relevant categories have been chosen. Where information is not explicit we tried to extract the minimum level of blinding. For placebo trials we have assumed that the patients are blinded.

Table S2: Detailed characteristics of the cohort

Category	No of Articles (% of all articles, n=2804)
**Research Location**	
N. America	1473 (52.53%)
Europe	695 (24.79%)
Unknown	393 (14.02%)
Australasia	211 (7.52%)
Asia	94 (3.35%)
n/a	62 (2.21%)
Africa	45 (1.6%)
S. America	32 (1.14%)
**Funding**	
Government	1418 (50.57%)
Unknown	961 (34.27%)
Non-commercial	502 (17.9%)
Commercial	119 (4.24%)
Not funded	43 (1.53%)
Other	4 (0.14%)
**Research Methods**	
Case report	1585 (56.53%)
Survey	505 (18.01%)
Qualitative interviews	259 (9.24%)
Systematic review and reviews	220 (7.85%)
Other	143 (5.1%)
Nested RCT	121 (4.32%)
Focus groups	108 (3.85%)
Vignettes	68 (2.43%)
Randomised study	18 (0.64%)
Workshop proceedings	11 (0.39%)
Nested case control	5 (0.18%)
Unknown	3 (0.11%)
**Research Outcomes**	
Numbers recruited	1483 (52.89%)
Other	597 (21.29%)
Reasons for participation or refusal	566 (20.19%)
Representativeness	444 (15.83%)
Willingness to participate	312 (11.13%)
Recruitment cost	159 (5.67%)
No evaluation	121 (4.32%)
Recruitment rate	57 (2.03%)
**Timing within host study**	
During study	1591 (56.74%)

N/A not nested in a study	806 (28.74%)
During feasibility	369 (13.16%)
Other	35 (1.25%)
Unknown	12 (0.43%)
**Host Design**	
RCT	1967 (70.15%)
Unknown	254 (9.06%)
Cohort	224 (7.99%)
Other	121 (4.32%)
Survey	104 (3.71%)
Early phase trials (not randomised)	69 (2.46%)
Cluster RCT	67 (2.39%)
Factorial RCT	47 (1.68%)
Biobank	44 (1.57%)
Case control	42 (1.5%)
Interview	33 (1.18%)
Patient preference	29 (1.03%)
Crossover RCT	27 (0.96%)
Focus group	4 (0.14%)
N-of-1	4 (0.14%)
**Recruitment Approach**	
Direct	1338 (47.72%)
Unknown	1167 (41.62%)
Indirect	601 (21.43%)
**Recruitment Setting**	
Secondary or tertiary care	1158 (41.3%)
Unknown	901 (32.13%)
Other	373 (13.3%)
Primary care	357 (12.73%)
Emergency care	85 (3.03%)
Intensive care	53 (1.89%)
School	52 (1.85%)
Dentist	14 (0.5%)
Pharmacy	12 (0.43%)
**Health Area**	
Cancer	706 (25.18%)
Unknown	406 (14.48%)
Mental Health	378 (13.48%)
Infection	239 (8.52%)
Cardiovascular	229 (8.17%)
Generic	172 (6.13%)
Reproduction	162 (5.78%)
Neurological	144 (5.14%)

Metabolic and endocrine	103 (3.67%)
Musculoskeletal	85 (3.03%)
Respiratory	70 (2.5%)
Stroke	69 (2.46%)
Renal	50 (1.78%)
Inflammatory and immune	47 (1.68%)
Injuries	45 (1.6%)
Oral and Gastro	37 (1.32%)
Other	32 (1.14%)
Eye	28 (1%)
Skin	21 (0.75%)
Blood	12 (0.43%)
Ear	9 (0.32%)
Congenital	4 (0.14%)
**Health intervention Type**	
Unknown	821 (29.28%)
Drug	675 (24.07%)
Behavioural	440 (15.69%)
N/A not intervention study	368 (13.12%)
Other	255 (9.09%)
Surgery	130 (4.64%)
Physical	129 (4.6%)
Care pathway	95 (3.39%)
Vaccine	90 (3.21%)
Screening	55 (1.96%)
Medical device	45 (1.6%)
Biomarkers	8 (0.29%)
Diagnostic	7 (0.25%)
Stem cell	2 (0.07%)
**Health intervention Aim**	
Treatment	1096 (39.09%)
Unknown	789 (28.14%)
Prevention	481 (17.15%)
N/A not intervention study	371 (13.23%)
Other	109 (3.89%)
Diagnostic	20 (0.71%)
**Health Intervention Setting**	
Unknown	1513 (53.96%)
Secondary or tertiary care	533 (19.01%)
N/A not an interventional study	372 (13.27%)
Other	160 (5.71%)
Primary care	142 (5.06%)
Emergency care	47 (1.68%)

Intensive care	44 (1.57%)
School	15 (0.53%)
Dentist	12 (0.43%)
Pharmacy	4 (0.14%)
**Age of recruited patients**	
Older than 18	1439 (51.32%)
Older than 60	979 (34.91%)
Unknown	953 (33.99%)
Younger than 16	332 (11.84%)
16-18 yrs	207 (7.38%)
**Gender recruited**	
Mixed	2277 (81.21%)
Female only	473 (16.87%)
Male only	115 (4.1%)
**Blinding within host study**	
Unknown	1474 (52.57%)
Open	500 (17.83%)
N/A not intervention study	415 (14.8%)
Patient and healthcare provider	261 (9.31%)
Patient only	193 (6.88%)
Health care provider only	3 (0.11%)

[a] Articles were coded against all applicable options. [b] Not all studies reported formal recruitment research outcomes e.g. case reports. Data reported in each article was reviewed to capture and categorise both formal and informal recruitment research outcomes. [c] Direct recruitment was the approach of patients known or likely to be eligible for inclusion. Indirect recruitment captured wider methods of approaching potential participants such as radio, newspaper adverts or community outreach. [d] Recruitment setting considered which healthcare settings were used in the identification or consent of patients. Where no health care setting (e.g. primary, secondary) were noted, but community outreach methods were described this was categorised as 'Other'. [e] Where articles were not explicit this was automatically categorised as mixed. [f] Blinding was poorly reported making categorisation tricky. Where unclear we categorised against the following assumptions: the use of placebo was listed as patient only based on the minimum level of blinding likely. Double blind was categorised as patient and healthcare provider unless there was some indication that it was only the patient and an end point assessor that was blinded. End point assessor blinding was not captured due to the unlikely effect on recruitment.

Table S3: Top ten journals publishing recruitment research

Journal	Frequency	Percentage (n=2804)
Clinical Trials (incl. supplements)	103	3.67
Contemporary Clinical Trials	102	3.64
Controlled Clinical Trials	79	2.82
Trials	77	2.74
Journal of Clinical Oncology (incl. supplements and annual meetings)	61	2.18
Journal of Clinical Epidemiology	54	1.93
Cancer	41	1.46
Annals of Epidemiology	33	1.18
Journal of the National Medical Association	28	1.00
Family Practice	27	0.96
British Journal of Cancer	25	0.89

Table S4: Distribution of individual domains against evidence categories

	Evidence Level			Overall
**Domain**	**1**	**2**	**3**	
**Trial Design**				
Blinding (A1)	8	1	9	18
Randomisation method (A2)	8	31	19	58
Opt in/ Opt out (A3)	10	13	18	41
Consent Timing (A4)	4	14	26	44
Patient/ Clinician Preference (A5)	7	13	23	43
Patient/ Clinician Convenience (A6)	1	6	5	12
**Pre Trial Planning**				
Participant acceptability of trial (B1)	8	44	348	400
Recruiter acceptability of trial (B2)	0	9	82	91
Feasibility studies (B3)	3	23	82	108
Participant Eligibility criteria (B4)	1	61	150	212
Trial Site Eligibility criteria (B5)	0	6	18	24
Sample size estimation (B6)	0	36	28	64
Recruitment rate prediction (B7)	4	47	81	132
Importance of outcomes to patients (B8)	0	3	7	10
Importance of out-comes to recruiters (B9)	0	1	1	2
Barriers and facilitators (B10)	7	42	425	474
**Trial Design and Conduct**				
Monitoring and systems (C1)	3	102	51	156
Administrative burden (C2)	1	29	65	95
Barriers and facilitators (C3)	10	171	886	1047
Trial Setting (C4)	1	75	118	194
Resources (C5)	5	149	155	309
Organisation (C6)	0	31	33	64
Patient Identification (C7)	8	334	379	721
Consent Process (C8)	26	76	200	302
Cultural considerations (C9)	11	106	331	448
**Recruitment information needs**				
Researcher training (D1)	5	22	40	67
Participant information (D2)	44	38	77	159
Delivery of information (D3)	53	146	82	281
Technology (D4)	21	67	23	111
Cultural considerations (D5)	11	45	90	146
Non- trial specific information (D6)	12	13	10	35
Trial Marketing (D7)	7	145	152	304
Reporting (D8)	1	3	4	8
**Recruiter Differences**				

Recruiter Engagement (E1)	5	68	175	248
Recruiter Characteristics (E2)	8	36	114	158
Format of data collection (E3)	10	5	6	21
Contact/ Engagement of recruiters and patients (E4)	4	28	86	118
Trial Site Characteristics (E5)	1	7	14	22
Recruiter Equipoise (E6)	0	8	32	40
**Incentives**				
Participant incentives (F1)	22	77	99	195
Recruiter incentives (F2)	6	33	39	78

Figure S1: Total website users, sessions and searches from September 2016 and May 2017

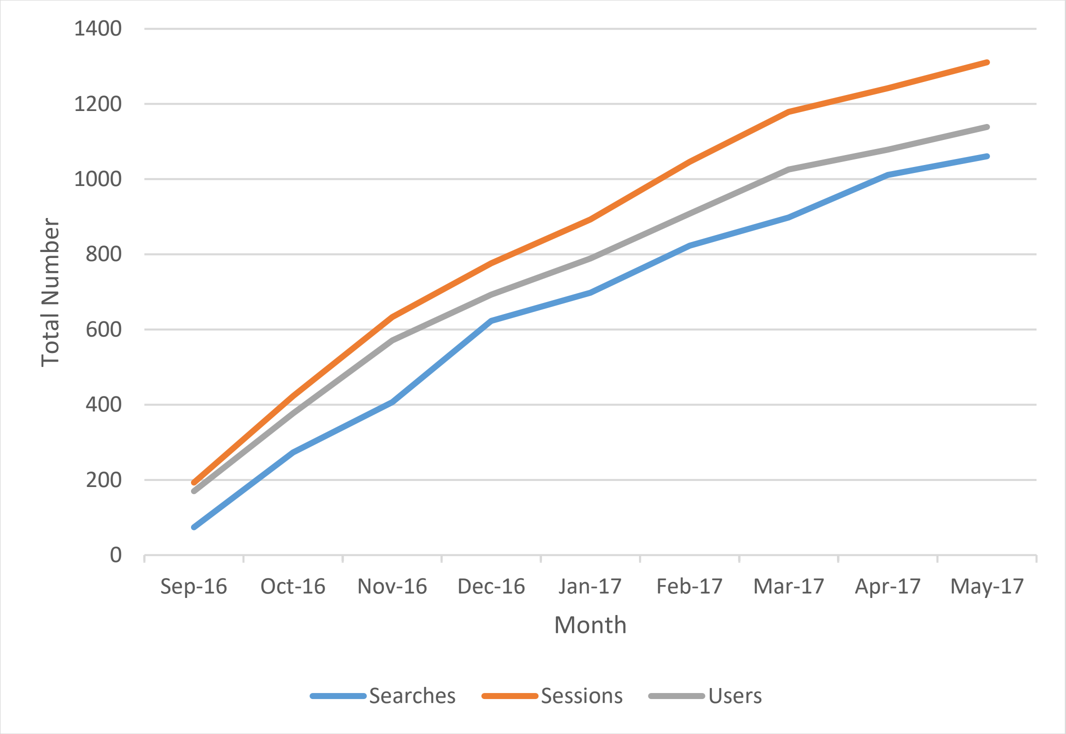

[1]184 Session /181 users were removed from the records for 05/12/16 and 251 sessions / 251 users were removed from the data on 11/04/17 as we believe these were generated from development and testing of updates. Filters were applied to google analytics from 13th December 2016 to improve the accuracy of user statistics and further changes were made in April 2017.

Table S5: Countries accessing ORRCA website between September 2016 and May 2017

Country	Sessions
1. United Kingdom[1]	1172
2. Ireland	51
3. United States	43
4. (not set)	16
5. Australia	6
6. Spain	4
7. Qatar	3
8. Nigeria	2
9. Belgium	2
10. Netherlands	2
11. India	2
12. Argentina	1

13. Canada	1
14. France	1
15. Hong Kong	1
16. Italy	1
17. Poland	1
18. Brazil	1
19. Sweden	1
**TOTAL**	**1311**

184 Session were removed from the records for 05/12/16 and 251 sessions were removed from the data on 11/04/17 as we believe these were generated from development and testing of updates. Filters were applied to google analytics from 13th December 2016 to improve the accuracy of user statistics and further changes were made in April 2017.

Table S6: Analysis of search terms used within the ORRCA database between September 2016 and May 2017

Search Field Heading	No of searches (n=1058*)
Recruitment domain(s)	374 (35%)
Text search	245 (23%)
Title	99 (9%)
Health area	54 (5%)
Author	41 (4%)
Recruitment approach	34 (3%)
Health intervention type	32 (3%)
Age	30 (3%)
Recruitment setting	29 (3%)
Host design	27 (3%)
Abstract	25 (2%)
Recruitment intervention	25 (2%)
Research location	23 (2%)
Research methods	20 (2%)
Year To	14 (1%)
Health intervention setting	14 (1%)
Year From	13 (1%)
Type of funding	10 (1%)
Evidence Type	9 (1%)
Health description	9 (1%)
Journal	6 (1%)
Health intervention aim	5 (<1%)
Blinding	5 (<1%)
Gender	3 (<1%)
Research outcomes	1 (<1%)
Volume	0 (0%)
Issue	0 (0%)
Pages	0 (0%)
DOI	0 (0%)
Summary of findings	0 (0%)
Timing within host study	0 (0%)

Notes: * A total of 1061 searches were recorded during the period. However, 3 searches were excluded from the analysis as they were unlikely to represent valid searches by users due to the use of randomly generated letter combinations across all available search fields.

Table S7: Frequency of searches for individual recruitment domains

Domains	1	2	3	4	5	6	7	8	9	10	Total No of domains (N=701*)
**A**	8	8	3	7	26	7					**59 (8%)**
**B**	25	15	15	7	11	21	154	6	4	58	**316 (45%)**
**C**	10	13	65	21	10	7	10	14	7		**157 (22%)**
**D**	12	7	4	19	7	2	8	5			**64 (9%)**
**E**	13	10	2	8	9	29					**71 (10%)**
**F**	17	17									**34 (5%)**
**G**	0										**0 (0%)**

Notes: *Individual searches could contain more than one domain. Initially website users could manually type in the domains as well as pre-selecting them from a drop down list. Subsequently the database was amended to only allow selection of domains from a dropdown menu. Incorrect free text searches have been removed from this analysis but remain in the overall figures.
